# Characteristics of Patients Using Patient-Facing Application Programming Interface Technology at a US Health Care System

**DOI:** 10.1001/jamanetworkopen.2020.22408

**Published:** 2020-10-27

**Authors:** William J. Gordon, Vaishali Patel, William Thornhill, David W. Bates, Adam Landman

**Affiliations:** 1 Division of General Internal Medicine, Brigham and Women’s Hospital, Boston, Massachusetts; 2 Office of the National Coordinator, Department of Health and Human Services, Washington, DC; 3 Mass General Brigham, Boston, Massachusetts; 4 Department of Emergency Medicine, Brigham and Women’s Hospital, Boston, Massachusetts

## Abstract

This case-control study investigates the characteristics of patients who use application programming interfaces connected to personal electronic devices to access electronic health information at a single US health care system.

## Introduction

For the purpose of increasing patients’ access to their electronic health information, the 21st Century Cures Act requires health information technology developers (such as electronic health record [EHR] vendors) to make available application programming interfaces (APIs), a mechanism of providing granular, computable access to data.^1^ The Office of the National Coordinator for Health Information Technology's Final Rule implementing the health information technology provisions of the 21st Century Cures Act was published in May 2020.^2^ Application programming interfaces allow patients to connect personal devices to the EHR to access their electronic health information, typically through an application, or app. The 21st Century Cures Act and its final rule are key milestones in improving patient access to electronic health information.^3^

Although early research shows slow but increasing use of apps,^4,5,6^ no studies to date have examined the clinical, health care utilization, and demographic characteristics of patients who have connected their personal devices to an EHR. Therefore, we sought to describe these characteristics among patients connecting apps to an EHR using APIs.

## Methods

We identified adult patients at a single institution (Mass General Brigham, Boston, Massachusetts) who had authorized an app to download their electronic health information from the EHR (API users) from January 1, 2019, through July 1, 2020. The API users were matched 1:3 with control patients who had used our portal at least once during the study period from a mobile device but had not connected an app using an API (API nonusers). The Mass General Brigham Institutional Review Board approved the study and waived individual consent because this retrospective research presented no more than minimal risk. This study followed the Strengthening the Reporting of Observational Studies in Epidemiology (STROBE) reporting guideline for case-control studies.

A portal login is required for connecting an app to the EHR using an API. We performed a multivariable logistic regression to compute odds ratios (ORs) with 95% CIs for the odds that a patient connected an app to our EHR. Model covariates included sex, age, race/ethnicity, language, and Charlson Comorbidity Index category—low (≤2), moderate (3-4), and high (≥5), calculated from *International Statistical Classification of Diseases, Tenth Revision, Clinical Modification (ICD-10-CM)* codes.

We performed unadjusted analyses to examine utilization differences between API users and API nonusers, including the number of inpatient encounters, outpatient encounters, emergency department encounters, prescribed medications, and unique clinicians seen during the study period. The Kruskal-Wallis test was used to assess intracategory differences. A 2-sided *P* ≤ .05 was considered statistically significant. Data analysis was conducted using R statistical software, version 3.5.1 (R Project for Statistical Computing).

## Results

Of the 10 895 API users identified, 6226 (57%) were male, and 10 593 (97%) were primary English speakers. Use of the API was, overall, low at 1% of our total patient portal population (roughly 1 million patients). Characteristics associated with an increased likelihood of being an API user included male sex (OR, 2.22; 95% CI, 2.13-2.33; *P* < .001) and younger age (OR, 0.98; 95% CI, 0.98-0.99; *P* < .001 for each year of age). Black, Hispanic, and White patients were less likely to be API users than Asian patients (Black patients: OR, 0.83; 95% CI, 0.73-0.95; Hispanic patients: OR, 0.75; 95% CI, 0.62-0.91; and White patients: OR, 0.91; 95% CI, 0.84-1.00), and Spanish-speaking patients were less likely to be API users than English-speaking patients (OR, 0.49; 95% CI, 0.37-0.64; *P* < .001). Moderate comorbidity scores were associated with a greater likelihood of using the APIs than low Charlson Comorbidity Index scores (Table 1). The API nonusers with at least 1 encounter tended to have more inpatient and unique clinician encounters than API users. No significant difference in emergency department encounters was observed (Table 2).

**Table 1.** Association of Baseline Patient Characteristics With API Use^a^

Characteristic	Unadjusted, No. (%)		Adjusted	
	API users (n = 10 895)	API nonusers (n = 31 931)	OR (95% CI)	*P* value
Sex				
Female	4669 (43)	19 414 (61)	1 [Reference]	
Male	6226 (57)	12 517 (39)	2.22 (2.13-2.33)	<.001
Age, median (IQR), y	42 (32-56)	46 (34-59)	0.98 (0.98-0.99)	<.001
Race/ethnic group				
Asian	793 (7)	1909 (6)	1 [Reference]	
Black	494 (5)	1535 (5)	0.83 (0.73-0.95)	.008
Hispanic	187 (2)	664 (2)	0.75 (0.62-0.91)	.003
White	8666 (80)	25 444 (80)	0.91 (0.84-1.00)	.046
Other or not available^b^	755 (7)	2379 (7)	0.83 (0.74-0.94)	.002
Language				
English	10 593 (97)	30 640 (96)	1 [Reference]	
Arabic	9 (<0.1)	64 (0.2)	0.43 (0.20-0.82)	.02
Chinese or Cantonese	4 (<0.1)	24 (<0.1)	0.54 (0.16- 1.45)	.27
Russian	9 (<0.1)	25 (<0.1)	1.18 (0.51-2.50)	.67
Spanish	65 (1)	413 (1)	0.49 (0.37-0.64)	<.001
Not available or other^c^	215 (2)	765 (2)	0.83 (0.71-0.97)	.02
Charlson Comorbidity Index score				
Low (≤2)	10 285 (94)	29 999 (94)	1 [Reference]	
Moderate (2-4)	494 (5)	1521 (5)	1.12 (1.00-1.25)	.04
High (≥5)	116 (1)	411 (1)	1.01 (0.81-1.24)	.96

Abbreviations: API, application programming interface; IQR, interquartile range; OR, odds ratio.

^a^ The API nonusers accessed our portal using a mobile device but did connect their device via an API.

^b^ Other denotes American Indian or Alaska Native, Native Hawaiian or other Pacific Islander, or not reported.

^c^ Other includes the following languages with at least 5 study participants: Portuguese, Korean, Vietnamese, American Sign Language, Haitian Creole, Japanese, Italian, and Albanian or not recorded.

**Table 2.** Unadjusted Health Care Utilization Characteristics of API Users Compared With API Nonusers^a^

Characteristic	No. (%)		*P* value^b^
	API users (n = 10 895)	API nonusers (n = 31 931)	
Inpatient encounters			
0	10 024 (92)	28 544 (89)	<.001
1-2	153 (1)	514 (2)	
2-5	495 (5)	2010 (6)	
6-10	132 (1)	571 (2)	
>10	91 (1)	292 (1)	
Outpatient encounters			
0	2080 (19)	4536 (14)	<.001
1-2	432 (4)	1006 (3)	
2-5	1014 (9)	2517 (8)	
6-10	1268 (12)	3490 (11)	
>10	6101 (56)	20381 (64)	
Unknown	0	1	
Emergency department encounters			
0	9669 (89)	28 351 (89)	>.99
1-2	719 (7)	2050 (6)	
2-5	373 (3)	1159 (4)	
6-10	99 (1)	289 (1)	
>10	35 (0.3)	82 (0.3)	
Medication orders			
0	3692 (34)	9479 (30)	<.001
1-2	452 (4)	1175 (4)	
2-5	1062 (10)	3004 (9)	
6-10	1244 (11)	3923 (12)	
>10	4445 (41)	14 350 (45)	
Unique clinicians			
0	6985 (64)	18 991 (59)	<.001
1-2	1615 (15)	5364 (17)	
2-5	1692 (16)	5816 (18)	
6-10	488 (4)	1465 (5)	
>10	115 (1)	295 (1)	

Abbreviation: API, application programming interface.

^a^ The API nonusers accessed our portal using a mobile device but did connect their device via an API.

^b^ Statistical tests performed: Kruskal-Wallis test.

## Discussion

Results of this case-control study indicate that patients who connected apps to our EHR using APIs were more likely to be male, younger, and speak English as their primary language than patients who did not use an app to connect to the EHR. Unadjusted differences in clinical utilization were less pronounced. Limitations include single-institution analysis and lack of information about utilization outside our institution. These trends, particularly the demographic differences, are important to monitor as policy efforts continue to expand patient access to electronic health information, so that all groups of patients can benefit from this technology.
